# Statistical Significance of the Maximum Hardness Principle Applied to Some Selected Chemical Reactions

**DOI:** 10.3390/molecules21111477

**Published:** 2016-11-05

**Authors:** Ranajit Saha, Sudip Pan, Pratim K. Chattaraj

**Affiliations:** Department of Chemistry and Centre for Theoretical Studies, Indian Institute of Technology Kharagpur, Kharagpur 721302, India; ranajitsahachem@gmail.com (R.S.); sudip.chem88@gmail.com (S.P.)

**Keywords:** density functional theory, maximum hardness principle, anomeric effect, disproportionation reactions

## Abstract

The validity of the maximum hardness principle (MHP) is tested in the cases of 50 chemical reactions, most of which are organic in nature and exhibit anomeric effect. To explore the effect of the level of theory on the validity of MHP in an exothermic reaction, B3LYP/6-311++G(2df,3pd) and LC-BLYP/6-311++G(2df,3pd) (def2-QZVP for iodine and mercury) levels are employed. Different approximations like the geometric mean of hardness and combined hardness are considered in case there are multiple reactants and/or products. It is observed that, based on the geometric mean of hardness, while 82% of the studied reactions obey the MHP at the B3LYP level, 84% of the reactions follow this rule at the LC-BLYP level. Most of the reactions possess the hardest species on the product side. A 50% null hypothesis is rejected at a 1% level of significance.

## 1. Introduction

The conceptual density functional theory (CDFT) [1,2] has been shown to be useful in analyzing popular concepts in chemistry like electronegativity (χ) [3,4,5,6], chemical hardness (η) [7,8,9,10], electrophilicity (ω) [11,12,13,14], etc. The idea of hardness was first introduced by Pearson in the context of his famous hard-soft acids and bases (HSAB) principle [7,10,15,16,17,18], which states that “hard acids prefer to coordinate with hard bases and soft acids prefer to coordinate with soft bases”. In 1987, Pearson proposed the maximum hardness principle (MHP) [19] as “there seems to be a rule of nature that molecules arrange themselves so as to be as hard as possible”. Now, for the ground state of an N electronic system, hardness (η) is defined as:

(1) $$\eta =\;{\left(\frac{\partial^{2}E}{\partial N^{2}}\right)}_{v\left(\vec{r}\right)}$$

where *E* is the total energy of the system, and this equation is valid for constant external potential ($v(\vec{r})$).

A finite difference approximation to Equation (1) gives

(2) η=(I−A)

where *I* and *A* are the ionization potential and electron affinity, respectively.

Now according to Koopmans’ theorem [20], *I* and *A* can be approximated in terms of the energies of the frontier molecular orbitals (*I* = *−E_HOMO_* and *A* = *−E_LUMO_*) and the Equation (2) can be modified as

(3) $$\eta =\left(E_{LUMO}-E_{HOMO}\right)$$

where *E_HOMO_* and *E_LUMO_* are the energies of the highest occupied molecular orbital (HOMO) and the lowest unoccupied molecular orbital (LUMO), respectively.

Thus, the stability of molecules, atoms or ions is connected to their HOMO-LUMO energy difference. Moreover, the favorable direction of a chemical reaction can also be understood by comparing the HOMO-LUMO energy gaps of the reactants and the products.

A statistical mechanical proof of the MHP was given by Parr and Chattaraj [21] in 1991. They showed that the MHP holds good under two conditions; (i) constant electronic chemical potential (μ) and (ii) constant external potential ($v(\vec{r})$). The validity of MHP has been checked for a wide range of physico-chemical processes and found to be valid in many situations like internal rotations [22,23,24,25,26,27,28], molecular vibrations [29,30,31,32], isomer stability [33], chemical reactions [34,35,36,37,38,39,40,41,42,43,44,45], Woodward-Hoffmann rules [46,47], aromaticity [48,49,50], stability of magic clusters [51], atomic shell structure [52,53], time-dependent situations [54,55,56], electronic excitations [57], chaotic ionizations [58], etc. There are also certain examples in literature where the MHP is not properly obeyed [59,60,61,62,63,64,65,66].

In an article, Poater et al. [67] studied 34 reactions given in the BH76 set, of which 28 are exothermic and the other 6 reactions are thermoneutral. They found that the products have greater hardness than the reactants in only 46% of the reactions, and this is reduced to only 18% of the total reactions upon the inclusion of another criterion, lower hardness in the transition state than that of the reactant. The reason may be that very hard atoms (like H, N, O, F, etc.) or molecules (like H_2_, N_2_, HF, HCN, CH_4_, etc.) were present on the reactant side. Recently, Pan et al. studied 101 chemical reactions to check the validity of the MHP in which most of them were inorganic reactions [68]. The study showed that the null hypothesis associated with the chemical reactions obeying the MHP is rejected at the 5% level of significance.

In this article, we are interested in checking whether the MHP is valid in some selected chemical reactions. For this purpose we have investigated 50 exothermic chemical reactions, the majority of which are organic, exhibiting a special steroelectronic effect called the anomeric effect [69,70,71], to analyze the validity of the MHP therein. Other than this we have studied some disproportionation reactions [72] in the light of the MHP. The effect of level of theory on the validity of the MHP in the studied set is also explored. For the reactions involving more than one reactant and/or product, we have computed the geometric mean (η_geo_) of the η values. Further, the combined hardness (η_com_) of the product or reactant side is also calculated as the difference between minimum ionization potential (*I*_min_) and maximum electron affinity (*A*_max_) values, respectively. The performance of these two approximations is compared in validating the MHP. Statistical testing of the null hypothesis is also performed in the validity of the MHP in the studied set of reactions.

## 2. Computational Details Section

Geometries of all of the studied molecules are modeled in the Gaussview 5.0.8 [73] graphical interface. The geometries are optimized at the DFT level of theory using two different functionals, the Becke three-parameter hybrid functional combined with Lee–Yang–Parr correlation, B3LYP [74,75] and long-range corrected LC-BLYP [75,76,77,78,79,80]. The 6-311++G(2df,3pd) [81,82] basis set is used for the calculations, except for iodine and mercury. For iodine and mercury, we have used the def2-QZVP [83] basis set along with effective core potential to take care of the relativistic effects. Frequency calculations with the optimized geometries are also carried out at the same level of theory. These frequency calculations are necessary to ensure that the optimized structures are at the minima of the respective potential energy surfaces. All of the above calculations are performed with the Gaussian 09 program package [84]. For these computations, we have used a fine grid, a pruned (75,302) grid with 75 radial shells per atom and 302 angular points per shell, which is the default in the Gaussian 09 program. Furthermore, the default values for convergence criteria of the self-consistent field and geometry are also used.

The *I* and *A* values using the Koopmans’ theorem and the η values using Equation (3) are calculated for each molecule appearing on the reactant and product sides. The combined hardness, η_com_ [30,85,86], is computed as:

η_com_ = (*I*_min_ − *A*_max_) (4)

where *I*_min_ and *A*_max_ are the minimum of the ionization potential and the maximum of the electron affinity values of the molecules appearing on the reactant or product side.

## 3. Results and Discussion

The computed values of *I* and *A* at the B3LYP/6-311++G(2df,3pd)/def2-QZVP and LC-BLYP/6-311++G(2df,3pd)/def2-QZVP levels for each molecule involved in the considered set of reactions are tabulated in Table S1 (in Supplementary Materials). It is found that *I* and *A* values differ considerably at these two levels. *I* values at the LC-BLYP level are found to be higher by 2.74–4.24 eV than those at the B3LYP level, whereas *A* values at the former level are lower by 1.06–3.95 eV than those at the latter level. Interestingly, while the *A* values are negative in all species studied here at the LC-BLYP level, they are positive at the B3LYP level. Therefore, not only do these two levels provide significantly different *I* and *A* values quantitatively, but in the case of *A* they also produce a qualitatively different trend. Such huge changes in *I* and *A* values led us to check whether they provide similar results in obeying the MHP, despite considerable changes in η values. Note that LC-BLYP functional was reported to be superior to B3LYP in predicting such properties. The computed η values of the reactants and products are presented in Table 1 and Table 2 obtained from the B3LYP and LC-BLYP levels, respectively. All of the reactions are exothermic in nature. Therefore, according to the MHP, the exothermic reactions are expected to have greater hardness on the product side as compared to the reactant side, considering the entropy effects to be negligible.

### 3.1. Results at the B3LYP Level

Among the 50 reactions considered here, 41 reactions and thus 82.0% of the total reactions obey the MHP when the geometric means of the η values are considered, while it is violated in nine reactions (see Table 1). Among these nine reactions, in two reactions (reaction number 29 and 45) the η_geo_ values in the reactant and product sides are almost same. For these nine reactions which do not follow the MHP, two major observations can be made: (1) In these reactions, the hardest species like CH_4_, CF_4_, CH_2_Cl_2_, and CH_3_F lie on the reactant side, and (2) species with very small η values are found on the product side, which makes the geometric mean of the η on the product side lower than that of the reactant side. However, the comparison of η_com_ between the reactants and products reduces the success of the MHP sharply. It is found that only 23 chemical reactions leading to just 46% of the total 50 reactions obey the MHP. It may be noted that in many cases the *I*_min_ and *A*_max_ are associated with the same reactant and/or product, which leads to the wrong trend. Therefore, for these cases we discourage the use of η_com_ to analyze the validity of the MHP. We have also tested the validity of the criterion that the hardest species lies on the product side. It is noted that only six reactions among the reactions in this set violate this criterion (88% of the total reactions obey). Interestingly, four of nine reactions, which previously disobeyed the MHP based on their η_geo_ values, follow this criterion. In one case (reaction number 34), although η_geo_ of products is larger than that of the reactants, the hardest species, (CH_3_)_2_CO, lies on the reactant side.

### 3.2. Results at the LC-BLYP Level

Despite large alteration in η values at the LC-BLYP level compared to those at B3LYP level, the number of reactions obeying the MHP does not change drastically. In fact, the total number of reactions obeying the MHP increases slightly at this level. 42 of the total 50 reactions are found to have η_geo_ values higher on the product side than that on the reactant side (see Table 2). Thus, 84% of the total reactions considered here are found to obey the MHP. Importantly, five of the nine reactions for which the MHP fails at the B3LYP level also give similar results at the LC-BLYP level. Similar to the previous level, here the use of the η_com_ value yields discouraging results where only 21 (i.e., 42%) reactions have η_com_ values higher on the product side than those on the reactant side. On the other hand, only eight reactions have the hardest species on the reactant side. Note that five of these eight reactions also contradict the MHP based on the η_geo_ values.

### 3.3. Test of the Null Hypothesis

Among the 50 reactions considered in Table 1, 41 reactions are found to obey the MHP at B3LYP/6-311++G(2df,3pd)/def2-QZVP level of theory. Thus, 82.0% reactions obey the MHP. Now we have tested the validity of the 50% null hypothesis for these set of reactions in Table 1. Let *P*_0_ be the population proportion of the chemical reactions mentioned above, and let us check the null hypothesis

H_0_: *P*_0_ = 0.5 (5)

against the alternative hypothesis,

H_1_: P_0_ ≠ 0.5 (6)

We have fixed the level of significance at 1% to check the null hypothesis. Thus, here the level of confidence is 99%. Note that the sample proportion (*P*) is binomial with mean *P*_0_ and variance *P*_0_(1 − *P*_0_)/n. Here, the sample size is 50, and the distribution of the test statistic calculated by the following expression,

(7) $$Z=\frac{\left(P-P_{0}\right)}{\sqrt{\frac{P_{0}(1-P_{0})}{n}}}$$

which may be approximated by the standard normal distribution in case the null hypothesis turns out to be true.

The null hypothesis will be rejected at the 1% level of significance if the absolute value of the calculated Z is greater than Z_0.005_(=2.578). The calculated value for Z is found to be 4.525, which is greater than Z_0.005_. Hence, the 50% null hypothesis is rejected at the 1% level of significance and it may be concluded that the proportion of reactions cannot be considered to be equal to 0.5.

Now let us combine the present set of organic type reactions with the previously reported inorganic-based reactions [68] to have a large set of total 151 reactions studied at the B3LYP/6-311++G(2df,3pd)/def2-QZVP level. Among these 151 reactions considered, 103 reactions have a geometric mean of the hardness of the products higher than the geometric mean of hardness of the reactants. Thus, 68.2% of reactions obey the MHP. Now we have tested the validity of 50% null hypothesis for the present sample size of 151 reactions. The Z-value is found to be 4.473, which is greater than Z_0.005_(2.578). Hence, the 50% null hypothesis is rejected at the 1% level of significance and it may be concluded that the proportion of reactions cannot be considered to be equal to 0.5.

Thus, the level of the significance remains more or less same in the case of the 50 reactions as well as the larger set of the reactions with a sample size of 151. Moreover, it is known from ref. [68] that the calculated hardness values differ significantly by changing the approximate formulas used, the quality of the basis set and the level of theory used. In the present cases, both the levels provide qualitatively similar trends regarding the validity of the MHP.

## 4. Conclusions

We have studied 50 exothermic reactions, most of which are organic in nature, exhibiting anomeric effect to investigate whether the maximum hardness principle (MHP) is valid. The geometric mean values of hardness and combined hardness of the reactants and the products are used. The calculations are carried out at the B3LYP/6-311++G(2df,3pd)/def2-QZVP and LC-BLYP/6-311++G(2df,3pd)/def2-QZVP (def2-QZVP for iodine and Hg) theory level to evaluate the effect of level of theory on the validation of the MHP in a given set of reactions. The results from the B3LYP/6-311++G(2df,3pd)/def2-QZVP level show that 82% of the studied reactions obey the MHP as they have a higher geometric mean of the hardness values on the product side compared to that on the reactant side. However, when the combined hardness is considered, only 46% of the chemical reactions obey the MHP at the same level of theory. The results at the LC-BLYP/6-311++G(2df,3pd)/def2-QZVP level are even marginally better. The geometric mean consideration shows that 84% of the chemical reactions obey the MHP, while the combined hardness values show that only 42% of the chemical reactions follow the MHP. At both levels, the number of reactions with the hardest species on the product side is reasonably high (44 at B3LYP and 42 at LC-BLYP out of 50 reactions). In the case where a total of 151 reactions are considered, the 50% null hypothesis is rejected at the 1% level of significance. Therefore, the validity of the MHP in so many reactions is not fortuitous.

## Figures and Tables

**Table 1:** Hardness (η, eV) values of the individual reactants and products, geometric mean (η_geo_, eV) of the hardness values and combined hardness values (η_com_, eV) for the reactant and product sides at the B3LYP/6-311++G(2df,3pd)/def2-QZVP (def2-QZVP for iodine and Hg) level for the chemical reactions. The enthalpy changes (∆H) for the reactions are in kcal/mol.

	Reactions							η_geo_(R)	η_geo_(P)	P > R?	η_com_(R)	η_com_(P)	P > R?	∆H ^#^
1.	2CH_3_F			→	CH_4_	+	CH_2_F_2_	9.49	10.12	Yes	9.49	9.64	Yes	−14 [69]
	9.49				10.62		9.64							
2.	2CH_3_OH			→	CH_4_	+	CH_2_(OH)_2_	7.41	9.02	Yes	7.41	7.66	Yes	−15 ± 5 [69]
	7.41				10.62		7.66							
3.	CH_3_F	+	CH_3_OH	→	CH_4_	+	FCH_2_OH	8.39	9.55	Yes	7.41	8.58	Yes	−ve [69]
	9.49		7.41		10.62		8.58							
4.	CH_3_NH_2_	+	CH_3_OH	→	CH_4_	+	HOCH_2_NH_2_	6.86	8.28	Yes	6.31	6.46	Yes	−ve [69]
	6.35		7.41		10.62		6.46							
5.	2CH_3_NH_2_			→	CH_4_	+	CH_2_(NH_2_)_2_	6.35	8.00	Yes	6.35	6.03	No	−ve [69]
	6.35				10.62		6.03							
6.	CH_3_NH_2_	+	CH_3_F	→	CH_4_	+	FCH_2_NH_2_	7.77	8.93	Yes	6.35	7.51	Yes	−ve [69]
	6.35		9.49		10.62		7.51							
7.	2SiH_3_F			→	SiH_4_	+	SiH_2_F_2_	8.94	9.47	Yes	8.94	9.30	Yes	−8 [69]
	8.94				9.54		9.41							
8.	2CF_2_Cl_2_			→	CF_4_	+	CCl_4_	7.84	9.22	Yes	7.84	6.76	No	−16.3 [69]
	7.84				12.59		6.76							
9.	3CH_3_F			→	2CH_4_	+	CHF_3_	9.49	10.78	Yes	9.49	10.62	Yes	−31.4 [69]
	9.49				10.62		11.11							
10.	4CHF_3_			→	CH_4_	+	3CF_4_	11.11	12.07	Yes	11.11	10.62	No	−22.9 [69]
	11.11				10.62		12.59							
11.	4CH_3_F			→	3CH_4_	+	CF_4_	9.49	11.08	Yes	9.49	10.62	Yes	−63 [69]
	9.49				10.62		12.59							
12.	4CH_3_Cl			→	3CH_4_	+	CCl_4_	7.81	9.49	Yes	7.81	6.76	No	−6 [69]
	7.81				10.62		6.76							
13.	4CH_3_OCH_3_			→	3CH_4_	+	C(OCH_3_)_4_	7.02	9.77	Yes	7.02	7.59	Yes	−52 [69]
	7.02				10.62		7.59							
14.	4CF_3_Cl			→	3CF_4_	+	CCl_4_	9.11	10.78	Yes	9.11	6.76	No	−27.1 [69]
	9.11				12.59		6.76							
15.	4CH_3_CH_3_			→	3CH_4_	+	C(CH_3_)_4_	9.25	10.04	Yes	9.25	8.47	No	−13 [69]
	9.25				10.62		8.47							
16.	4SiH_3_F			→	3SiH_4_	+	SiF_4_	8.94	10.04	Yes	8.94	8.85	No	−23 [69]
	8.94				9.54		11.72							
17.	SiF_3_H	+	CF_4_	→	SiF_4_	+	CF_3_H	11.83	11.41	No	11.11	10.44	No	−37 [69]
	11.11		12.59		11.72		11.11							
18.	C(OCH_3_)_4_	+	SiH_4_	→	CH_4_	+	Si(OCH_3_)_4_	8.51	8.98	Yes	7.59	7.60	Yes	−144 [69]
	7.59		9.54		10.62		7.59							
19.	2CH_3_OH			→	H_2_O	+	(CH_3_)_2_O	7.41	7.60	Yes	7.41	6.64	No	−6.0 [72]
	7.41				8.23		7.02							
20.	2HOF			→	H_2_O	+	F_2_O	7.09	7.39	Yes	7.09	5.82	No	−5 ± 3 [72]
	7.09				8.23		6.63							
21.	2HOCl			→	H_2_O	+	Cl_2_O	5.99	5.90	No	5.99	4.22	No	−1 ± 1 [72]
	5.99				8.23		4.22							
22.	2CH_3_SH			→	H_2_S	+	(CH_3_)_2_S	6.11	6.28	Yes	6.11	5.55	No	−2.8 [72]
	6.11				6.78		5.82							
23.	2HSSH			→	H_2_S	+	(HS)_2_S	4.37	6.09	Yes	4.37	5.47	Yes	−4.4 [72]
	4.37				6.78		5.47							
24.	2CH_3_NH_2_			→	NH_3_	+	(CH_3_)_2_NH	6.35	6.47	Yes	6.35	5.77	No	−4.5 [72]
	6.35				7.05		5.94							
25.	2(CH_3_)_2_NH			→	(CH_3_)_3_N	+	CH_3_NH_2_	5.94	6.01	Yes	5.94	5.65	No	−2.4 [72]
	5.94				5.69		6.35							
26.	2CH_3_CH_3_			→	CH_4_	+	(CH_3_)_2_CH_2_	9.25	9.67	Yes	9.25	8.80	No	−2.5 [72]
	9.25				10.62		8.80							
27.	2CF_3_H			→	CH_2_F_2_	+	CF_4_	11.11	11.02	No	11.11	9.64	No	−0 ± 2 [72]
	11.11				9.64		12.59							
28.	2CH_3_Cl			→	CH_4_	+	CH_2_Cl_2_	7.81	9.03	Yes	7.81	7.68	No	−1.5 ± 1.4 [72]
	7.81				10.62		7.68							
29.	CH_2_Cl_2_	+	CCl_4_	→	2CHCl_3_			7.20	7.19	No	6.43	7.19	Yes	−2.7 ± 1 [72]
	7.68		6.76		7.19									
30.	CH_4_	+	CH_2_I_2_	→	2CH_3_I			7.21	5.77	No	4.89	5.77	Yes	−4.4 [72]
	10.62		4.89		5.77									
31.	2H(CH_3_)C=CH_2_			→	H_2_C=CH_2_	+	(CH_3_)_2_C=CH_2_	6.94	6.98	Yes	6.94	5.56	No	−1.3 [72]
	6.94				7.40		6.60							
32.	2HFC=CF_2_			→	H_2_C=CF_2_	+	F_2_C=CF_2_	7.37	7.42	Yes	7.37	7.34	No	−4.5 ± 4 [72]
	7.37				7.50		7.34							
33.	H_2_C=CCl_2_	+	Cl_2_C=CCl_2_	→	2HClC=CCl_2_			6.12	6.74	Yes	5.82	6.74	Yes	−2 ± 4 [72]
	6.43		5.82		6.74									
34.	H_2_CO	+	(CH_3_)_2_CO	→	2CH_3_CHO			6.10	6.25	Yes	5.31	6.25	Yes	−1.7 ± 1.5 [72]
	5.94		6.26		6.25									
35.	COCl_2_	+	(CH_3_)_2_CO	→	2Cl(CH_3_)CO			6.61	6.98	Yes	4.98	6.98	Yes	−13.5 [72]
	6.98		6.26		6.98									
36.	(CH_3_)_2_Hg	+	Cl_2_Hg	→	2MeHgCl			6.11	6.79	Yes	3.94	6.79	Yes	−12 ± 3 [72]
	6.39		5.84		6.79									
37.	2COS			→	CO_2_	+	CS_2_	7.24	7.32	Yes	7.24	5.38	No	0.0 [72]
	7.24				9.95		5.38							
38.	2CH_2_CO			→	CO_2_	+	CH_2_=C=CH_2_	5.66	8.58	Yes	5.66	7.00	Yes	−27 ± 2 [72]
	5.66				9.95		7.40							
39.	2CH_2_CO			→	CO_2_	+	CH_4_	5.66	10.28	Yes	5.66	7.24	Yes	−60 ± 2 [72]
	5.66				9.95		10.62							
40.	2CH_2_CS			→	CS_2_	+	CH_4_	4.01	7.56	Yes	4.01	5.38	Yes	−30 ± 4 [72]
	4.01				5.38		10.62							
41.	2COCl_2_			→	CO_2_	+	CCl_4_	6.98	8.20	Yes	6.98	6.76	No	−12 [72]
	6.98				9.95		6.76							
42.	2COF_2_			→	CO_2_	+	CF_4_	9.26	11.20	Yes	9.26	9.95	Yes	−14 ± 4 [72]
	9.26				9.95		12.59							
43.	HC(OH)_3_			→	HCOOH	+	H_2_O	8.11	7.99	No	8.11	7.75	No	−7 [72]
	8.11				7.75		8.23							
44.	SiH_3_Cl	+	CH_3_F	→	SiH_3_F	+	CH_3_Cl	8.78	8.36	No	8.63	7.56	No	−20 [70]
	8.13		9.49		8.94		7.81							
45.	SiH_3_PH_2_	+	CH_3_NH_2_	→	SiH_3_NH_2_	+	CH_3_PH_2_	6.62	6.60	No	6.17	6.46	Yes	−16 [70]
	6.91		6.35		6.68		6.53							
46.	SiH_3_SiH_3_	+	CH_3_CH_3_	→	2SiH_3_CH_3_			8.54	8.67	Yes	7.89	8.67	Yes	−7 [70]
	7.89		9.25		8.67									
47.	SiH_3_I	+	HOF	→	SiH_3_F	+	HOI	6.65	6.02	No	5.45	4.05	No	−80 [70]
	6.25		7.09		8.94		4.05							
48.	2CH_2_F_2_			→	CHF_3_	+	CH_3_F	9.64	10.27	Yes	9.64	9.49	No	−8.5 [71]
	9.64				11.11		9.49							
49.	2NH_2_F			→	NHF_2_	+	NH_3_	7.51	7.69	Yes	7.51	7.01	No	−10.0 [71]
	7.51				8.39		7.05							
50.	2NHF_2_			→	NF_3_	+	NH_2_F	8.39	8.66	Yes	8.39	7.51	No	−6.0 [71]
	8.39				9.99		7.51							

^#^ The reference numbers from where the enthalpy values are taken are given in the square brackets. Hardness values (in eV) are provided below each molecule.

**Table 2:** Hardness (η, eV) values of the individual reactants and products, geometric mean (η_geo_, eV) of the hardness values and combined hardness values (η_com_, eV) for the reactant and product sides at the LC-BLYP/6-311++G(2df,3pd)/def2-QZVP (def2-QZVP for iodine and Hg) level of theory for the chemical reactions. The enthalpy changes (∆H) for the reactions are in kcal/mol.

	Reactions							η_geo_(R)	η_geo_(P)	P > R?	η_com_(R)	η_com_(P)	P > R?	∆H ^#^
1.	2CH_3_F			→	CH_4_	+	CH_2_F_2_	14.10	14.72	Yes	14.10	14.21	Yes	−14 [69]
	14.10				15.22		14.21							
2.	2CH_3_OH			→	CH_4_	+	CH_2_(OH)_2_	12.08	13.81	Yes	12.08	12.52	Yes	−15 ± 5 [69]
	12.08				15.22		12.52							
3.	CH_3_F	+	CH_3_OH	→	CH_4_	+	FCH_2_OH	13.05	14.24	Yes	12.08	13.32	Yes	−ve [69]
	14.10		12.08		15.22		13.32							
4.	CH_3_NH_2_	+	CH_3_OH	→	CH_4_	+	HOCH_2_NH_2_	11.42	12.93	Yes	10.77	10.98	Yes	−ve [69]
	10.80		12.08		15.22		10.98							
5.	2CH_3_NH_2_			→	CH_4_	+	CH_2_(NH_2_)_2_	10.80	12.62	Yes	10.80	10.47	No	−ve [69]
	10.80				15.22		10.47							
6.	CH_3_NH_2_	+	CH_3_F	→	CH_4_	+	FCH_2_NH_2_	12.34	13.58	Yes	10.80	12.11	Yes	−ve [69]
	10.80		14.10		15.22		12.11							
7.	2SiH_3_F			→	SiH_4_	+	SiH_2_F_2_	13.99	14.19	Yes	13.99	14.03	Yes	−8 [69]
	13.99				14.03		14.34							
8.	2CF_2_Cl_2_			→	CF_4_	+	CCl_4_	14.01	15.33	Yes	14.01	13.16	No	−16.3 [69]
	14.01				17.85		13.16							
9.	3CH_3_F			→	2CH_4_	+	CHF_3_	14.10	15.41	Yes	14.10	15.22	Yes	−31.4 [69]
	14.10				15.22		15.78							
10.	4CHF_3_			→	CH_4_	+	3CF_4_	15.78	17.16	Yes	15.78	15.22	No	−22.9 [69]
	15.78				15.22		17.85							
11.	4CH_3_F			→	3CH_4_	+	CF_4_	14.10	15.84	Yes	14.10	15.22	Yes	−63 [69]
	14.10				15.22		17.85							
12.	4CH_3_Cl			→	3CH_4_	+	CCl_4_	12.41	14.68	Yes	12.41	13.16	Yes	−6 [69]
	12.41				15.22		13.16							
13.	4CH_3_OCH_3_			→	3CH_4_	+	C(OCH_3_)_4_	11.48	14.68	Yes	11.48	12.12	Yes	−52 [69]
	11.48				15.22		12.12							
14.	4CF_3_Cl			→	3CF_4_	+	CCl_4_	14.89	16.54	Yes	14.89	13.16	No	−27.1 [69]
	14.89				17.85		13.16							
15.	4CH_3_CH_3_			→	3CH_4_	+	C(CH_3_)_4_	13.65	14.60	Yes	13.65	12.89	No	−13 [69]
	13.65				15.22		12.89							
16.	4SiH_3_F			→	3SiH_4_	+	SiF_4_	13.99	14.79	Yes	13.99	13.89	No	−23 [69]
	13.99				14.03		17.31							
17.	SiF_3_H	+	CF_4_	→	SiF_4_	+	CF_3_H	16.90	16.53	No	16.00	15.77	No	−37 [69]
	16.00		17.85		17.31		15.78							
18.	C(OCH_3_)_4_	+	SiH_4_	→	CH_4_	+	Si(OCH_3_)_4_	13.04	13.58	Yes	12.12	12.11	No	−144 [69]
	12.12		14.03		15.22		12.11							
19.	2CH_3_OH			→	H_2_O	+	(CH_3_)_2_O	12.08	12.41	Yes	12.08	11.40	No	−6.0 [69]
	12.08				13.42		11.48							
20.	2HOF			→	H_2_O	+	F_2_O	13.54	13.81	Yes	13.54	1.42	No	−5 ± 3 [69]
	13.54				13.42		14.22							
21.	2HOCl			→	H_2_O	+	Cl_2_O	11.79	12.02	Yes	11.79	10.76	No	−1 ± 1 [69]
	11.79				13.42		10.76							
22.	2CH_3_SH			→	H_2_S	+	(CH_3_)_2_S	10.58	10.68	Yes	10.58	9.95	No	−2.8 [69]
	10.58				11.36		10.04							
23.	2HSSH			→	H_2_S	+	(HS)_2_S	11.27	11.19	No	11.27	11.02	No	−4.4 [69]
	11.27				11.36		11.02							
24.	2CH_3_NH_2_			→	NH_3_	+	(CH_3_)_2_NH	10.80	11.00	Yes	10.80	10.26	No	−4.5 [69]
	10.80				11.80		10.26							
25.	2(CH_3_)_2_NH			→	(CH_3_)_3_N	+	CH_3_NH_2_	10.26	10.37	Yes	10.26	9.96	No	−2.4 [69]
	10.26				9.97		10.80							
26.	2CH_3_CH_3_			→	CH_4_	+	(CH_3_)_2_CH_2_	13.65	14.15	Yes	13.65	13.14	No	−2.5 [69]
	13.65				15.22		13.14							
27.	2CF_3_H			→	CH_2_F_2_	+	CF_4_	15.78	15.93	Yes	15.78	14.21	No	−0 ± 2 [69]
	15.78				14.21		17.85							
28.	2CH_3_Cl			→	CH_4_	+	CH_2_Cl_2_	12.41	13.87	Yes	12.41	12.64	Yes	−1.5 ± 1.4 [69]
	12.41				15.22		12.64							
29.	CH_2_Cl_2_	+	CCl_4_	→	2CHCl_3_			12.90	12.88	No	12.64	12.88	Yes	−2.7 ± 1 [72]
	12.64		13.16		12.88									
30.	CH_4_	+	CH_2_I_2_	→	2CH_3_I			12.53	10.74	No	10.31	10.74	Yes	−4.4 [72]
	15.22		10.31		10.74									
31.	2H(CH_3_)C=CH_2_			→	H_2_C=CH_2_	+	(CH_3_)_2_C=CH_2_	11.24	11.42	Yes	11.24	10.82	No	−1.3 [72]
	11.24				12.06		10.82							
32.	2HFC=CF_2_			→	H_2_C=CF_2_	+	F_2_C=CF_2_	11.88	12.02	Yes	11.88	11.81	No	−4.5 ± 4 [72]
	11.88				11.94		12.11							
33.	H_2_C=CCl_2_	+	Cl_2_C=CCl_2_	→	2HClC=CCl_2_			11.43	11.47	Yes	11.12	11.47	Yes	−2 ± 4 [72]
	11.49		11.36		11.47									
34.	H_2_CO	+	(CH_3_)_2_CO	→	2CH_3_CHO			11.53	11.51	No	11.14	11.51	Yes	−1.7 ± 1.5 [72]
	11.94		11.14		11.51									
35.	COCl_2_	+	(CH_3_)_2_CO	→	2Cl(CH_3_)CO			12.12	12.40	Yes	11.14	12.40	Yes	−13.5 [72]
	13.19		11.14		12.40									
36.	(CH_3_)_2_Hg	+	Cl_2_Hg	→	2MeHgCl			11.32	12.04	Yes	9.89	12.04	Yes	−12 ± 3 [72]
	10.79		11.88		12.04									
37.	2COS			→	CO_2_	+	CS_2_	12.63	12.55	No	12.63	10.49	No	0.0 [72]
	12.63				15.02		10.49							
38.	2CH_2_CO			→	CO_2_	+	CH_2_=C=CH_2_	11.01	13.25	Yes	11.01	11.69	Yes	−27 ± 2 [72]
	11.01				15.02		11.69							
39.	2CH_2_CO			→	CO_2_	+	CH_4_	11.10	15.12	Yes	11.02	12.41	Yes	−60 ± 2 [72]
	11.01				15.02		15.22							
40.	2CH_2_CS			→	CS_2_	+	CH_4_	9.49	12.64	Yes	9.49	10.49	Yes	−30 ± 4 [72]
	9.49				10.49		15.22							
41.	2COCl_2_			→	CO_2_	+	CCl_4_	13.19	14.06	Yes	13.19	13.16	No	−12 [72]
	13.19				15.02		13.16							
42.	2COF_2_			→	CO_2_	+	CF_4_	15.09	16.38	Yes	15.09	15.02	No	−14 ± 4 [72]
	15.09				15.02		17.85							
43	HC(OH)_3_			→	HCOOH	+	H_2_O	12.83	13.15	Yes	12.83	12.72	No	−7 [72]
	12.83				12.89		13.42							
44.	SiH_3_Cl	+	CH_3_F	→	SiH_3_F	+	CH_3_Cl	13.62	13.18	No	12.96	12.41	No	−20 [70]
	13.16		14.10		13.99		12.41							
45.	SiH_3_PH_2_	+	CH_3_NH_2_	→	SiH_3_NH_2_	+	CH_3_PH_2_	10.97	11.04	Yes	10.80	10.74	No	−16 [70]
	11.14		10.80		11.33		10.74							
46.	SiH_3_SiH_3_	+	CH_3_CH_3_	→	2SiH_3_CH_3_			13.07	13.20	Yes	12.14	13.20	Yes	−7 [70]
	12.14		13.65		13.20									
47.	SiH_3_I	+	HOF	→	SiH_3_F	+	HOI	12.38	11.72	No	11.20	9.82	No	−80 [70]
	11.31		13.54		13.99		9.82							
48.	2CH_2_F_2_			→	CHF_3_	+	CH_3_F	14.21	14.92	Yes	14.21	14.10	No	−8.5 [71]
	14.21				15.78		14.10							
49.	2NH_2_F			→	NHF_2_	+	NH_3_	12.24	12.42	Yes	12.24	11.74	No	−10.0 [71]
	12.24				13.07		11.80							
50.	2NHF_2_			→	NF_3_	+	NH_2_F	13.07	14.09	Yes	13.07	12.24	No	−6.0 [71]
	13.07				16.22		12.24							

^#^ The reference numbers from where the enthalpy values are taken are given within the square brackets. Hardness values (in eV) are provided below the molecules.

## Supplementary Materials

The following are available online at http://www.mdpi.com/1420-3049/21/11/1477/s1. The calculated ionization potential (I, eV) and electron affinity (A, eV) at B3LYP/6-311++G(2df,3pd)/def2-QZVP and LC-BLYP/6-311++G(2df,3pd)/def2-QZVP levels for the molecules are provided in the supplementary materials.
