# Supplementary material for: Statistical Significance of the Maximum Hardness Principle Applied to Some Selected Chemical Reactions
Source: Molecules. 2016 Nov 5;21(11):1477. doi: 10.3390/molecules21111477 (PMC6273755; doi:10.3390/molecules21111477)
Supplement: Supplementary file 1 [file molecules-21-01477-s001.pdf]

Supplementary Materials: Statistical Significance of the Maximum Hardness Principle Applied to Some Selected Chemical Reactions

Ranajit Saha, Sudip Pan and Pratim K. Chattaraj

Table S1. The Ionization Potential (I, eV) and Electron Affinity (A, eV) calculated at B3LYP/6-311++G(2df,3pd)/def2-QZVP and LC-BLYP/6-311++G(2df,3pd)/def2-QZVP levels for the molecules.

Molecule	B3LYP/6-311++G(2df,3pd)/def2-QZVP		LC-BLYP/6-311++G(2df,3pd)/def2-QZVP	
	Ionization Potential (I)	Electron Affinity (A)	Ionization Potential (I)	Electron Affinity (A)
CH_3F	9.68	0.18	13.22	−0.88
CH_4 (12.62)	10.77	0.15	14.31	−0.91
CH_2F_2	9.82	0.18	13.31	−0.89
CH_3OH	7.76	0.34	11.26	−0.81
$CH_2(OH)_2$	8.36	0.69	11.94	−0.59
FCH_2OH	9.02	0.44	12.53	−0.78
CH_3NH_2	6.66	0.30	9.96	−0.84
$HOCH_2NH_2$	6.91	0.45	10.24	−0.74
$CH_2(NH_2)_2$	6.48	0.45	9.73	−0.74
FCH_2NH_2	7.82	0.32	11.24	−0.87
SiH_3F	9.64	0.69	12.95	−1.04
SiH_2F_2	9.79	0.37	13.10	−1.24
SiH_4	9.67	0.13	12.98	−1.05
CF_2Cl_2	9.25	1.41	12.66	−1.35
CF_4	12.39	−0.20	16.25	−1.60
CCl_4	8.87	2.11	12.19	−0.97
CHF_3	11.26	0.14	14.86	−0.92
CH_3Cl	8.26	0.45	11.60	−0.81
CH_3OCH_3	7.23	0.22	10.60	−0.88
$C(OCH_3)_4$	7.74	0.15	11.24	−0.87
CF_3Cl	9.84	0.73	13.43	−1.47
CH_3CH_3	9.42	0.16	12.73	−0.92
$C(CH_3)_4$	8.78	0.31	12.06	−0.83
SiF_4	12.53	0.82	16.40	−0.91
SiF_3H	11.11	0.01	14.61	−1.39
$Si(OCH_3)_4$	7.83	0.23	11.26	−0.85
Me_2O	7.23	0.22	10.60	−0.88
H_2O	8.82	0.59	12.62	−0.80
H_2S	7.32	0.54	10.57	−0.79
MeHS	6.62	0.51	9.78	−0.81
$(HS)_2S$	7.32	1.85	10.31	−0.70
HSSH	6.23	1.85	10.47	−0.79
HSF	7.29	2.22	10.54	−0.54
HOF	9.12	2.04	12.79	−0.76
HOCl	8.02	2.04	11.37	−0.43
Cl_2O	8.10	3.87	11.35	0.58
F_2O	9.63	3.00	13.28	−0.95
NH_3	7.47	0.42	10.94	−0.86
$MeNH_2$	6.66	0.30	9.96	−0.84
Me_2NH	6.19	0.25	9.41	−0.85
NMe_3	5.95	0.27	9.11	−0.85
$HMeC{=}CH_2$	7.14	0.20	10.32	−0.91
$H_2C{=}CH_2$	7.69	0.29	10.98	−1.08
$Me_2C{=}CH_2$	6.85	0.25	9.97	−0.85

$F_2C{=}CF_2$	7.53	0.19	10.82	−1.29
$HFC{=}CF_2$	7.60	0.23	10.88	−1.00
$H_2C{=}CF_2$	7.65	0.15	10.95	−0.99
$Cl_2C{=}CCl_2$	7.20	1.38	10.15	−1.21
$HClC{=}CCl_2$	7.41	0.67	10.53	−0.93
$H_2C{=}CCl_2$	7.44	1.01	10.53	−0.97
Me_2Hg	6.81	0.42	9.96	−0.83
Cl_2Hg	8.70	2.85	11.95	0.07
MeHgCl	8.04	1.25	11.46	−0.57
COS	8.45	1.21	11.50	−1.13
CO_2	10.48	0.52	14.01	−1.01
CS_2	7.62	2.24	10.36	−0.13
$CH_2{=}C{=}CH_2$	7.53	0.13	10.72	−0.97
$CH_2{=}C{=}O$	6.95	1.29	10.11	−0.91
COF_2	10.20	0.94	13.90	−1.19
$COCl_2$	9.04	2.06	12.37	−0.82
CH_2CS	6.44	2.43	9.30	−0.19
Me_2S	6.09	0.27	9.16	−0.88
Me_2CH_2	8.99	0.18	12.23	−0.92
CH_2Cl_2	8.55	0.87	11.83	−0.82
CH_2I_2	7.17	2.28	10.03	−0.28
CH_3I	7.08	1.31	10.09	−0.66
$CHCl_3$	8.68	1.49	11.98	−0.90
H_2CO	7.66	1.72	11.04	−0.91
Me_2CO	7.03	0.78	10.44	−0.70
HMeCO	7.33	1.08	10.71	−0.79
ClMeCO	8.42	1.44	11.66	−0.73
HOI	7.16	3.11	10.18	0.35
SiH_3Cl	8.81	0.69	12.08	−1.09
SiH_3NH_2	7.21	0.53	10.48	−0.86
SiH_3PH_2	7.40	0.49	10.29	−0.85
SiH_3SiH_3	8.18	0.28	11.16	−0.97
SiH_3CH_3	8.99	0.32	12.32	−0.88
SiH_3I	7.48	1.23	10.45	−0.87
CH_3PH_2	6.99	0.47	9.94	−0.81
NH_2F	8.05	0.54	11.46	−0.77
NHF_2	8.86	0.47	12.26	−0.80
NF_3	10.22	0.23	13.65	−2.57
HCOOH	8.40	0.65	11.92	−0.97
$HC(OH)_3$	8.55	0.44	12.04	−0.79
